# Mapping the intellectual landscape and emerging trends of retinitis pigmentosa research

**DOI:** 10.3389/fmed.2026.1740088

**Published:** 2026-03-04

**Authors:** Li Xiao, Li-Xuan Tang, Min Ai, Yi-Jing Yang, Ying Deng, Jing Lu, Ya-Sha Zhou, Jing Xiong, Qinghua Peng

**Affiliations:** 1 Hunan University of Chinese Medicine, Changsha, Hunan, China; 2 Yueyang Hospital of Traditional Chinese Medicine, Yueyang, Hunan, China

**Keywords:** retinitis pigmentosa, bibliometric analysis, gene therapy, oxidative stress, retinal organoid

## Abstract

**Purpose:**

To visualize the intellectual landscape of retinitis pigmentosa (RP) research, outline recent advances, identify emerging trends, and offer insights into future directions.

**Methods:**

A bibliometric analysis was conducted on RP-related publications retrieved from the Web of Science and PubMed databases (retrieved on 12 December 2024) using CiteSpace and VOSviewer. The analysis focused on visualizing key dimensions, including annual publication volume, highly cited publications, and keyword distribution.

**Results:**

Analysis of 8,121 publications identified four main evolutionary stages: early epidemiological characterization, foundational genetic research, a transition toward clinical trials, and recent therapeutic innovation. Initial studies focused on disease mechanisms, whereas current research is largely oriented toward treatment. The field has become increasingly interdisciplinary, with close links among genetics, bioengineering, nanomedicine, and artificial intelligence-based diagnostics contributing to progress. Keyword burst analysis showed sustained research interest through 2024 in topics such as “optical coherence tomography angiography”, “retinal organoid”, and “cell death”. In addition, “gene therapy” and “oxidative stress” emerged as highly active and rapidly developing research themes.

**Conclusion:**

RP research is progressing quickly, with a clear emphasis on therapeutic strategies. The integration of multiple scientific disciplines has accelerated the development of new treatment approaches. Among these, gene therapy and antioxidant-based interventions show strong potential, particularly when used in combination, and represent promising directions for future breakthroughs in RP management.

## 1 Introduction

Retinitis pigmentosa (RP) is a genetically diverse retinopathy characterized by progressive degeneration of photoreceptor cells and atrophy of the retinal pigment epithelium, ultimately leading to bilateral blindness. This genetic heterogeneity poses significant challenges in RP research, making it difficult to develop universal treatments and highlighting the necessity for personalized therapies tailored to individual genetic profiles. The global prevalence of RP worldwide ranges between 1 in 7,000 and 1 in 3,000 individuals, making it one of the leading causes of irreversible blindness (1). RP significantly affects individuals’ lives and work, posing a substantial economic burden on families and society; however, despite ongoing advancements in medical research, critical knowledge gaps persist regarding its exact pathogenesis and the translational potential of emerging therapies. While genetic and molecular studies have expanded our understanding, systematic evaluations of research trends, particularly in treatment strategies and interdisciplinary approaches, remain underexplored. In light of these challenges, visualizing recent advancements and emerging research trends in RP becomes imperative. This approach can identify research priorities, foster collaborative networks, and accelerate the development of new treatments.

As a literature-driven research method, bibliometric analysis allows for the visualization of key research hotspots, emerging trends, and potential directions in a field of study. This method has been widely adopted across academic and practical domains (2–4), facilitating scientific innovation and the advancement of various disciplines. Bibliometric analysis can decode a field’s intellectual structure by tracking citation networks and co-authorship patterns, highlight understudied areas, and assess translational bottlenecks between preclinical and clinical research. For example, this approach has been instrumental in mapping advancements in neurodegenerative diseases and cardiovascular disease, clarifying evolving therapeutic paradigms and collaborative networks (5, 6). By analyzing key metrics such as publication output and citation count, bibliometric analysis objectively evaluates the academic contributions of researchers and institutions (7). It also supports the formulation of science and technology policies (8), offering data-driven insights for strategic decision-making. Additionally, in the context of discipline development, bibliometric analysis helps to identify research priorities, assess the level of academic progress, and guide resource allocation and talent cultivation (9). Moreover, it serves as a valuable tool in technology consulting by offering enterprises insights into the latest technological advancements and market trends, enhancing their innovation capacity (10).

While traditional narrative reviews are valuable, they can be inherently subjective and may not fully capture the complex and dynamic evolution of a scientific field. In contrast, bibliometric analysis offers an objective, quantitative, and macro-level perspective. It is particularly effective at revealing hidden patterns, assessing the influence of research themes, and identifying emerging frontiers that may be missed in conventional reviews. Tools such as CiteSpace (11) and VOSviewer (12) play an important role in this process. They use advanced algorithms, including co-occurrence analysis to map intellectual structures and burst detection to uncover early signals of new trends. This computational approach relies on specific metrics, such as node centrality, density, and betweenness, to evaluate the influence of research topics and the relationships among them. Importantly, this methodological framework goes beyond the basic keyword retrieval functions of search engines like PubMed. Although PubMed is highly effective for locating relevant studies through MeSH terms and title or abstract screening, it does not have the algorithmic capacity to explain the underlying knowledge structure of a field or how it changes over time. Bibliometric analysis therefore complements traditional retrieval methods by uncovering structural patterns, collaborative networks, and evolutionary pathways that simple search results cannot show.

In this study, we conducted a comprehensive bibliometric analysis of RP research using a combination of CiteSpace, VOSviewer, NoteExpress, and Excel, tools suited for network visualization and citation analysis. By examining factors such as publication volume, highly cited publications, and keyword distribution, we aim to provide a systematic overview of the research landscape and emerging trends in RP. This analysis is expected to provide valuable reference points for future academic investigations, contributing to ongoing progress and innovation in this field.

## 2 Methods

This study employed a bibliometric analysis to map the intellectual landscape and evolving trends in RP research. The analysis was conducted on all eligible scientific publications on RP retrieved from the target database. Using the software CiteSpace and VOSviewer, we examined key bibliometric indicators, including publication volume and temporal trends (productivity), citation counts (impact), as well as keyword co-occurrence networks, burst detection and high-frequency keyword analysis (conceptual structure and emerging trends). The study design is a retrospective, observational, and descriptive analysis of the existing literature corpus.

### 2.1 Data collection

To enhance the representativeness and accessibility of the data, we systematically searched the Web of Science (WoS) Core Collection and PubMed. All data were retrieved from these databases on 12 December 2024. The search strategy for WoS was as follows: TS = (“retinitis pigmentosa” OR “pigmentary retinopathy” OR “retinal pigment degeneration”). For PubMed, the search strategy was as follows: (“retinitis pigmentosa” [MeSH Major Topic]). No restrictions were placed on publication date, language, or document type.

### 2.2 Eligibility criteria

Studies were included if they focused on RP, covering one or more aspects of clinical diagnosis, treatment, prevention, basic experimental and biological studies. The following exclusion criteria were applied: (1) duplicate records; (2) publications unrelated to RP; and (3) records lacking essential information such as author, keywords, year of publication, which are critical for analysis.

### 2.3 Data analysis

**FIGURE 1:**
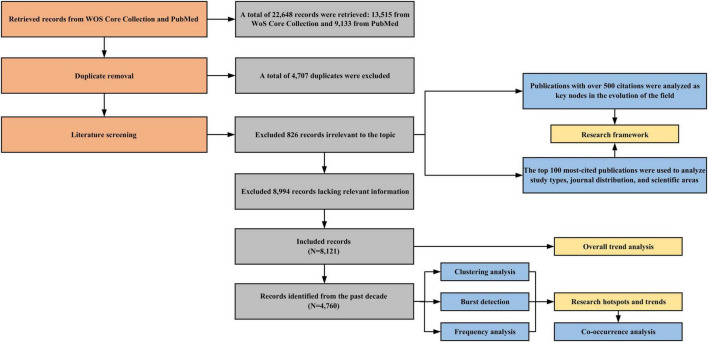
Flow diagram of the study selection process.

All retrieved data were downloaded and imported into NoteExpress 4.1.0 for duplicate removal. Two independent medical researchers screened the studies based on the predefined eligibility criteria. Any discrepancies were resolved through discussion, and if necessary, a third-party ophthalmologist provided a final decision. After screening, 8,121 studies were included. The literature selection process is illustrated in Figure 1. The final dataset was exported to Excel to analyze publication volume and the characteristics of highly cited publications, including study type, journal distribution, and scientific area. To conduct a systematic analysis of the conceptual structure and emerging trends, CiteSpace 6.3.R1 and VOSviewer 1.6.20 were used. These tools were selected for their advanced capabilities in network visualization and citation analysis, which are essential for deconstructing complex bibliographic relationships beyond the sorting functions of standard search engines.

Specifically, cluster analysis was used to group related keywords, which helped identify major research hotspots and the evolutionary paths of different sub-fields. The specific parameters set in CiteSpace were as follows: Timespan 2014–2024 with Slice Length set to 1; Selection Criteria g-index (*k* = 10), LRF = 2.5, L/N = 10, LBY = 5, and *e* = 1.0; Network *N* = 264 and *E* = 1,429 with a Density of 0.0412; Largest 1 CCs 255 (96%); Nodes Labeled 1.0%; Pruning none; Modularity *Q* = 0.3474; Weighted Mean Silhouette *S* = 0.6463; and Harmonic Mean (*Q*, *S*) = 0.4519. At the same time, burst detection was applied to track keywords showing sharp increases in citation frequency. This measure is important for identifying emerging frontiers and capturing early signals of new scientific directions before they become widely recognized. In addition, co-occurrence analysis was performed to generate density maps that visualize the relationships between gene therapy and oxidative stress. Together, these methods move beyond simple descriptive statistics and help clarify the evolutionary trajectory and potential mechanistic links within the RP research landscape.

## 3 Results

### 3.1 Development trend analysis

**FIGURE 2:**
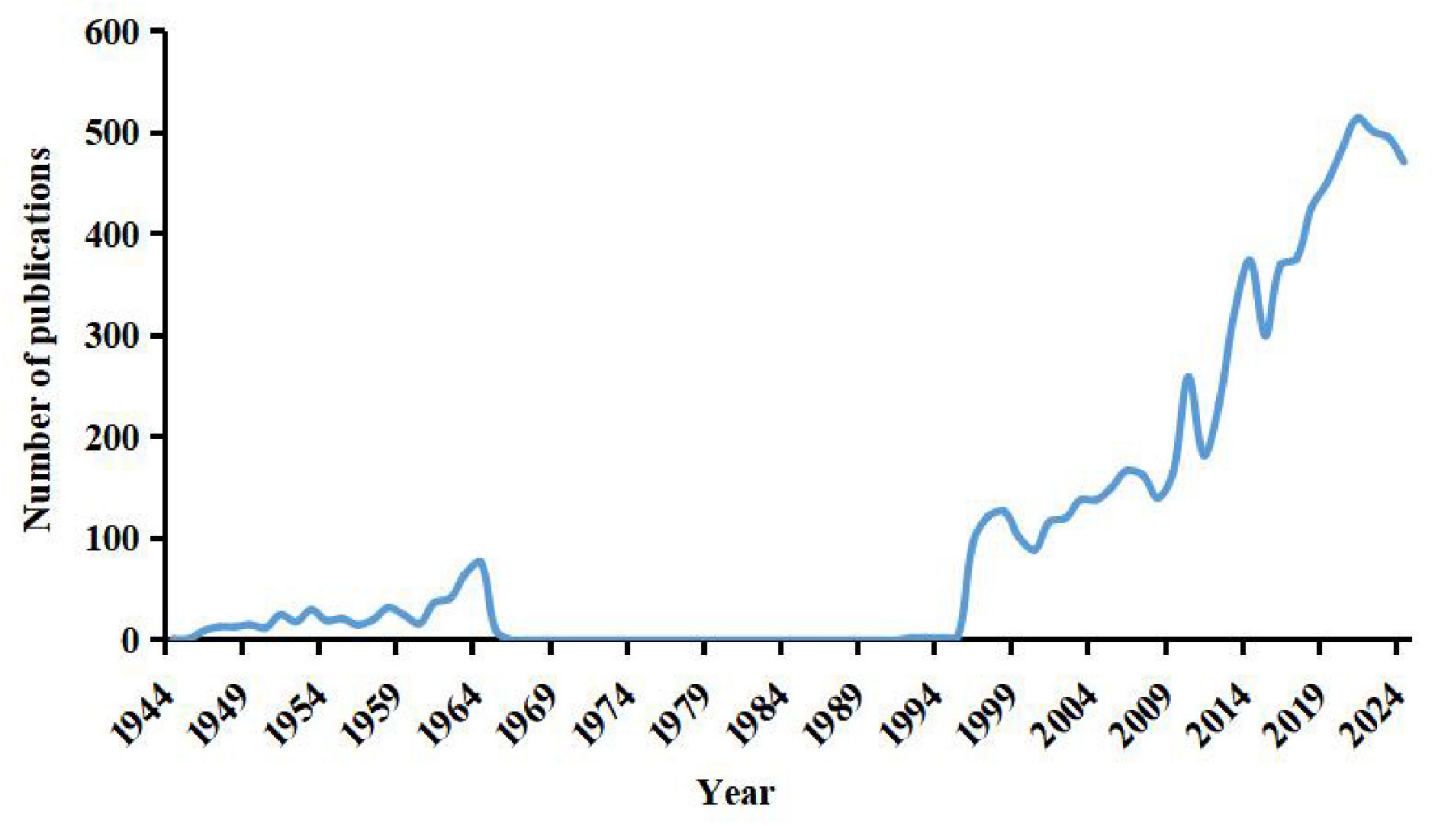
Annual number of publications on annual number of publications on retinitis pigmentosa.

Publication volume is a fundamental indicator of research productivity (13). The development trends in RP research were analyzed by examining annual publication volumes (Figure 2). As shown in Figure 2, the number of publications on RP has steadily increased over time, reflecting growing interest in this field. According to publication volume, the evolution of RP research can be divided into four distinct phases: (1) Initial stage (1944–1964): This period marked the early exploration of RP, with limited publications available. (2) Slow exploration stage (1965–1995): Research activity increased gradually, with most publications consisting of case reports. Due to insufficient data, these publications were largely excluded from keyword analysis. (3) Steady growth stage (1996–2013): Research in this phase reached momentum, leading to a more structured development of the field. (4) Rapid development stage (2014–present): Since 2014, RP research has significantly expanded, driven by advancements in biomedical technology and therapeutic approaches.

### 3.2 Highly cited publication analysis

**FIGURE 3:**
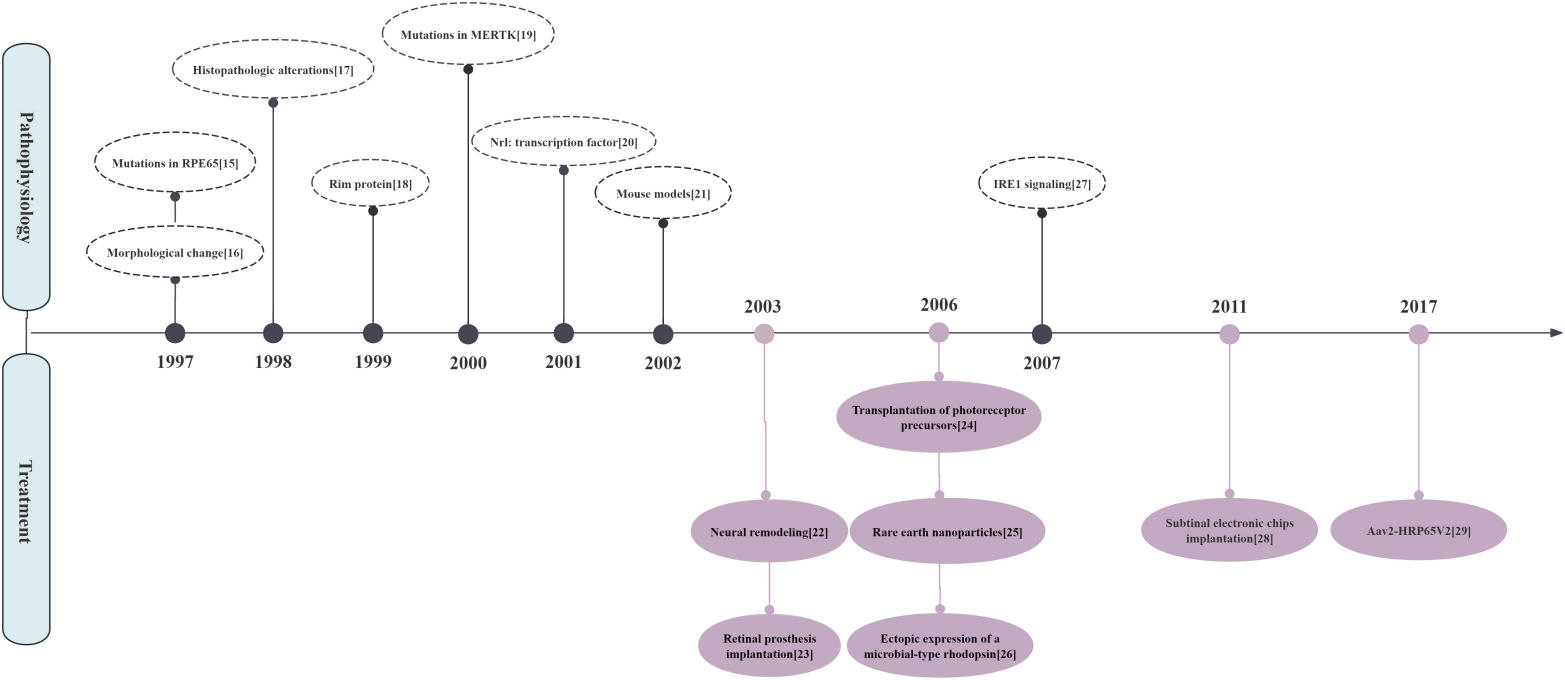
Evolution diagram of important retinitis pigmentosa themes (15–29).

Citation count is a key indicator of a study’s impact and contributions to the academic community (14). It functions as a quantitative reference for scientific significance and reflects the extent to which a study shapes later research. We confirmed that highly cited studies carry strong scientific value through direct content analysis. Such literature helps pinpoint milestones and shifts in research focus within RP studies. Figure 3 highlights publications with more than 500 citations, revealing a transition in research focus from pathogenesis in earlier studies to treatment approaches in recent years (15–29).

**FIGURE 4:**
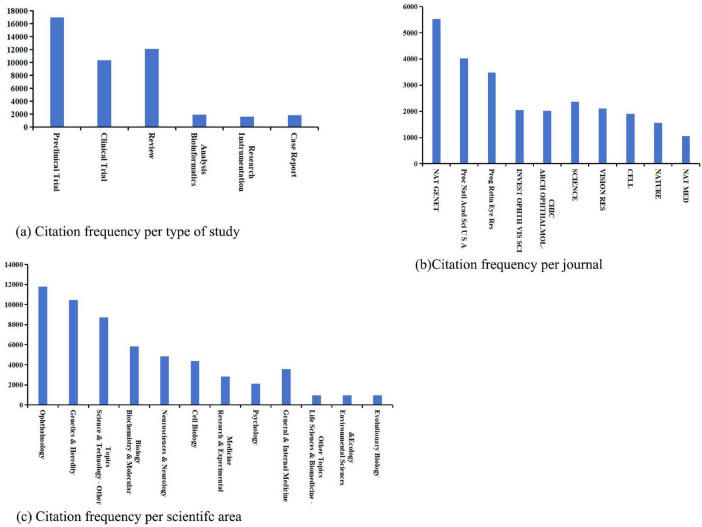
Bar charts illustrating the citation landscape of retinitis pigmentosa research across **(a)** study types, **(b)** journals, and **(c)** scientific areas, showing the top-ranked entries for each category.

A detailed analysis of the top 100 most-cited articles was conducted based on research types, journals, and scientific fields (Figure 4). This analysis aimed to identify dominant study types, influential journals, and trends in interdisciplinary collaboration. The primary research areas in this field include preclinical studies, such as cellular and animal experiments, and clinical trials, focusing on therapeutic interventions. Among academic journals, Nature Genetics (NAT GENET) has published a substantial number of high-impact publications on RP. Research in this field spans multiple scientific disciplines, reflecting its broad and interdisciplinary nature, with emerging synergies between genetics, bioengineering (e.g., retinal prosthetics), nanomedicine (e.g., drug delivery systems), and artificial intelligence-based diagnostics driving recent advancements. This interdisciplinary convergence has accelerated the development of innovative therapeutic strategies, such as CRISPR-based gene editing and stem cell therapies, while enabling high-throughput screening of potential drug candidates through computational approaches.

### 3.3 Research hotspots and trend analysis

**FIGURE 5:**
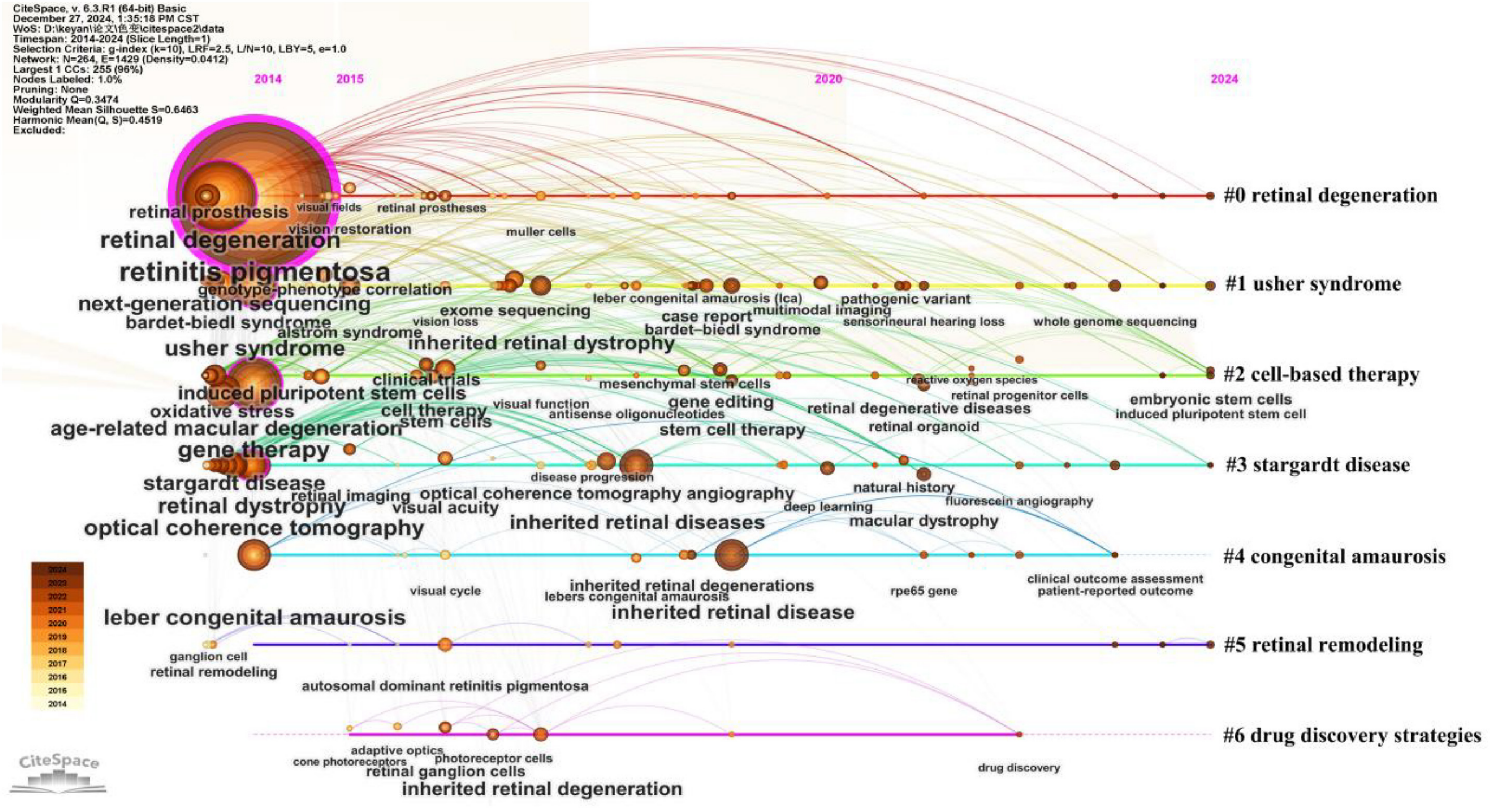
Timeline visualization of co-occurrence keyword analysis.

To identify emerging research hotspots and trends in RP, we conducted an in-depth keyword analysis of literature published over the past decade. This included clustering analysis, burst detection, and tracking high-frequency keyword trends. The clustering analysis results, displayed in a timeline view (Figure 5), illustrate the major research clusters and their evolution over time. RP research is broadly categorized into two primary areas: disease understanding (shown as clusters #0 retinal degeneration, #1 usher syndrome, #3 stargardt disease, #4 congenital amaurosis) and treatment approaches (shown as clusters #2 cell-based therapy, #5 retinal remodeling, #6 drug discovery strategies). The emergence of these clusters is closely tied to the dominant technologies of each research stage. Clusters #0 retinal degeneration and #1 usher syndrome reflect the Genomic Era, driven by the introduction of Next-Generation Sequencing, which required more precise disease classification. In contrast, cluster #2 cell-based therapy and cluster #5 retinal remodeling reflect the Regenerative Era, supported by advances in stem cell research and bioengineering that address the limits of gene-focused strategies in late-stage disease. This pattern shows that research clusters tend to form around the tools available at each stage of field development.

**FIGURE 6:**
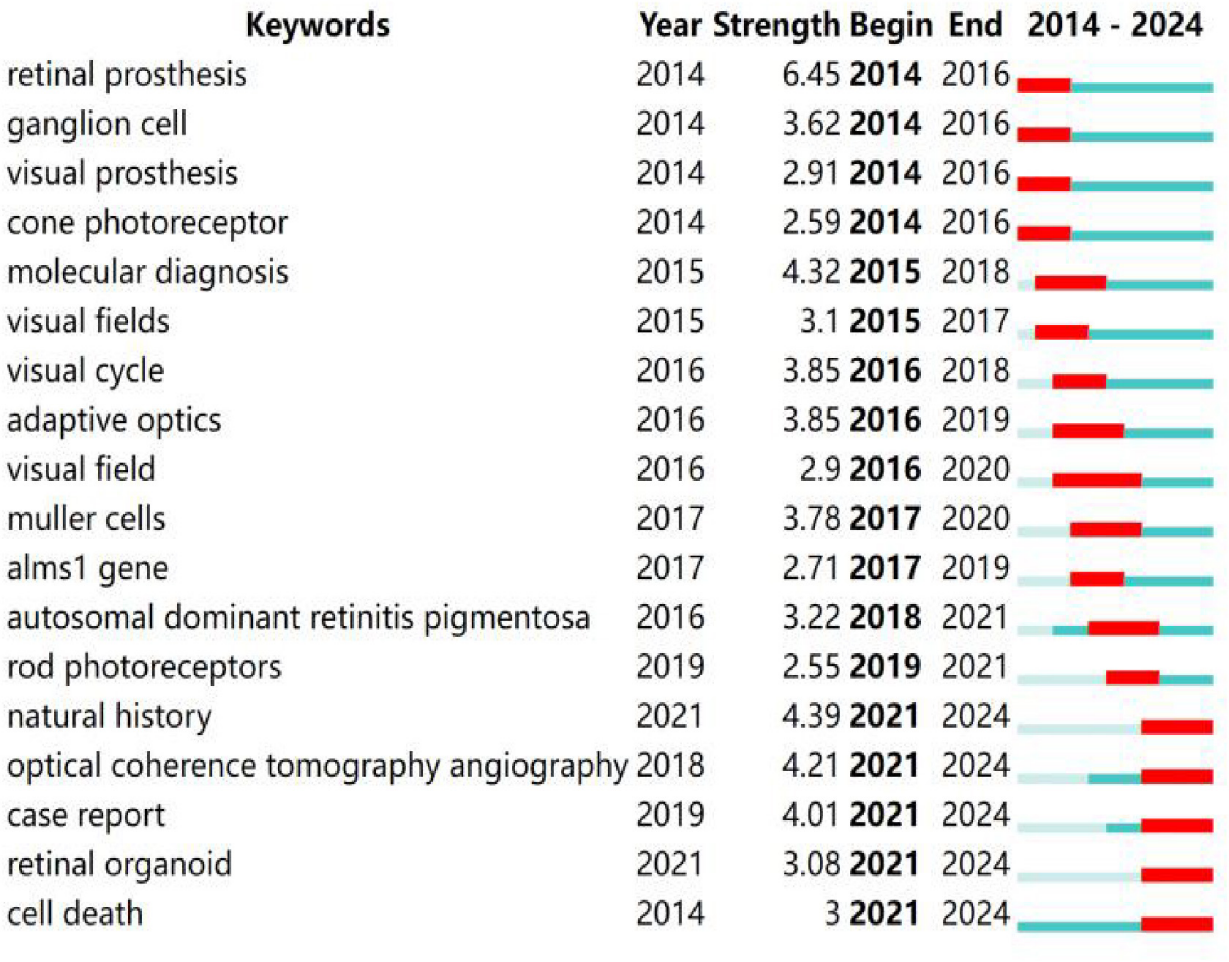
Top 18 keywords with the strongest citation bursts.

Burst detection identifies emerging topics with rapidly increasing research activity (11, 30). Figure 6 highlights keywords with strong bursts continuing through 2024, including “natural history,” “optical coherence tomography angiography (OCTA),” “case report,” “retinal organoid” and “cell death.” The rise of these keywords is driven by clear practical needs. The strong burst of OCTA reflects demand from clinical trials for sensitive and non-invasive biomarkers that can measure retinal capillary loss in real time, which goes beyond what traditional fundus photography can provide. Likewise, the growing focus on “retinal organoid” research stems from the need to move past the limits of animal models. Induced pluripotent stem cell technology now supports human-specific drug screening and aligns with the broader shift toward personalized treatment and functional recovery.

**FIGURE 7:**
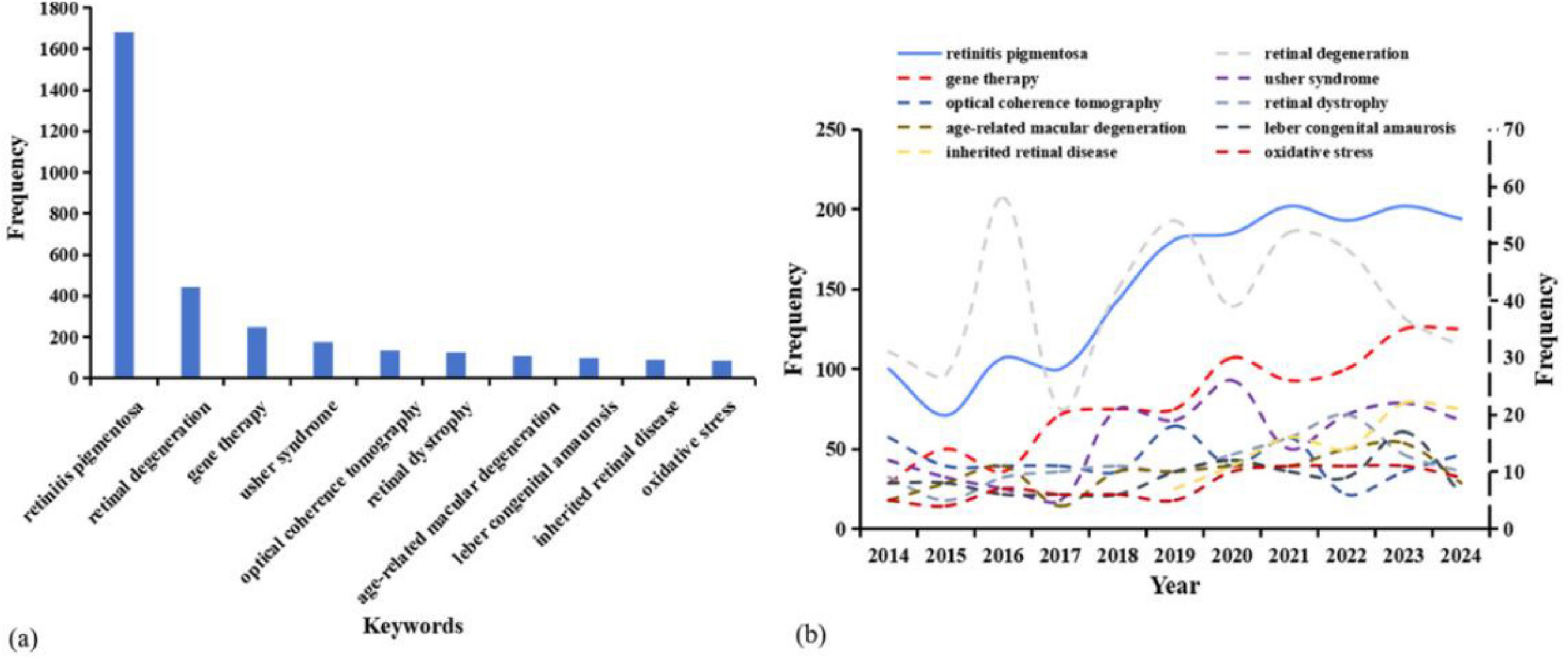
Analysis chart of high-frequency keywords **(a)** frequency sorting, **(b)** annual frequency trend.

**FIGURE 8:**
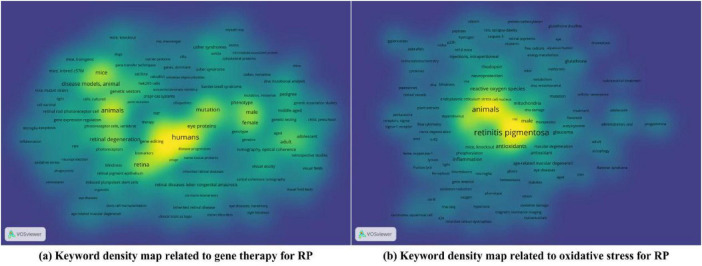
Density map of high-frequency keywords related to **(a)** gene therapy, **(b)** oxidative stress in the field of retinitis pigmentosa (RP).

Figure 7 displays the top 10 most frequently occurring keywords, along with trends in their usage over time. The analysis reveals that “gene therapy” and “oxidative stress” are not only hot topics in RP research but also exhibit an upward trend, highlighting their growing significance in the field. To explore how these themes are connected, we conducted a keyword co-occurrence density analysis centered on “gene therapy” and “oxidative stress” in Figures 8a, b. The density map for “gene therapy” in Figure 8a shows strong co-occurrence with terms such as “animals”, “humans”, “clinical trials”, as well as “antioxidants” and “oxidative stress”. This pattern suggests that research extends beyond basic concepts and covers the full path from experimental models to clinical use. The strong association between “gene therapy” and “antioxidants” points to a growing interest in supportive treatment strategies. It suggests a growing recognition in the field that the long-term efficacy of therapy relies not only on restoring wild-type gene function but also on modulating the disease microenvironment, specifically by targeting oxidative stress, to achieve disease modification (31). At the same time, the density map for “oxidative stress” in Figure 8b shows close links with “antioxidants”, “ferroptosis”, “inflammation”, and “gene deletion”. In this map, “ferroptosis” and “inflammation” appear alongside “oxidative stress” and “antioxidants”, forming a clear disease and treatment-related subnetwork. From a mechanistic perspective, genetic defects lead to metabolic imbalance and accumulation of reactive oxygen species. This triggers oxidative stress, ferroptosis, and subsequent inflammation through innate immune responses. This interconnected process highlights the need to target oxidative stress, ferroptosis, and inflammation together when designing combination therapies for RP. The frequent appearance of “gene deletion” also reflects the continued reliance on genetic models to confirm these specific molecular targets.

## 4 Discussion

Through a comprehensive bibliometric analysis that integrates publication volume trends, highly cited publication analysis, keyword timeline mapping, burst detection, and keyword co-occurrence density visualization, this study outlines the macro-level knowledge landscape of RP research. The findings point to a clear structural transition from fragmented description toward integrated intervention. This evolution can be summarized through three closely connected themes.

### 4.1 Evolution of research drivers and paradigms

Bibliometric evidence shows that the main driving force of RP research has shifted from early phenomenological observation to intervention-driven innovation. This transition is supported by annual publication trends and changes in the focus of highly cited studies. The shift has been shaped by a combined technology and policy environment. On the technology side, major advances such as CRISPR/Cas9 and adeno-associated virus vectors have played a central role. On the policy side, regulatory milestones, including the approval of Luxturna, have further accelerated progress. The increasing maturity and precision of CRISPR/Cas9 have made it routine to build high-fidelity disease models and perform targeted gene editing, which has greatly shortened the path from basic discovery to preclinical research (32, 33). At the same time, ongoing improvements in the safety, delivery efficiency, and immunogenicity of adeno-associated virus vectors have supported the clinical translation of gene-based therapies (34). In parallel, regulatory events such as the FDA approval of voretigene neparvovec in 2017 opened dedicated review pathways for orphan drugs and gene therapies. This reduced barriers to market entry and encouraged closer collaboration among academic researchers, industry partners, and clinicians (35). As a result, the rapid growth of RP research reflects not just rising academic interest, but a convergence of technical readiness, supportive regulation, and focused investment.

Consistent with this trend, our analysis of journals, research types, and disciplinary backgrounds within highly cited publications suggests that RP research is moving toward a more integrated scientific structure. Interdisciplinary collaboration in this field is no longer a simple layering of different disciplines. Instead, it has developed into a convergent research model that is strongly oriented toward clinical problems. For example, the development of retinal prostheses requires the combined input of microelectronic engineering, neuroscience, materials science, and surgical practice. Overall, the RP research landscape has evolved into a diverse, collaborative, and solution-focused innovation environment. The time-based patterns seen in highly cited publications and keyword clusters also point to a deeper paradigm change. Earlier work followed a reductionist logic centered on single genes or pathways. More recent studies emphasize integrative strategies aimed at systemic disease repair. Early milestone discoveries, such as RPE65 (15) and MERTK (19)] established the genetic foundation of RP but largely remained descriptive in nature. In contrast, recent years have seen a clear shift toward intervention, reflected by clustered advances in cell transplantation and retinal prosthetics. This change is closely linked to the highly heterogeneous nature of RP, where no single target or therapy can address all patient groups. As a result, there is growing agreement that effective management requires a multi-dimensional intervention strategy that combines genetic repair, cellular stress modulation, and functional replacement. This also explains the increasing influence of bioengineering, materials science, and data science in the field.

This paradigm shift has also reshaped research methods and ways of thinking. Earlier studies mainly focused on individual genes and their roles in RP pathogenesis. Current research places more weight on interactions among multiple genes and on the complex regulatory networks involved in disease progression. For instance, omics techniques—such as proteomics and metabolomics—provide insights into alterations at specific molecular levels in RP (36, 37). These approaches collectively contribute to a more comprehensive understanding of the molecular mechanisms underlying the disease. In addition, progress in systems biology has introduced new perspectives by treating the retina as a complex biological system. From this viewpoint, disease onset and progression are explored through interactions between the system and its environment. These integrative strategies help identify promising therapeutic targets and support the development of more effective treatment options.

### 4.2 Emerging frontiers and key tools

Burst detection analysis effectively identifies rapidly expanding research frontiers that address key bottlenecks in the field. The results indicate that “optical coherence tomography angiography” and “retinal organoids” have emerged as the fastest-growing research tools in recent years. Their increasing use directly reflects two major challenges in current RP research and clinical practice. One is the lack of methods for dynamic disease monitoring, and the other is the shortage of physiologically relevant experimental models. OCTA enables non-invasive and quantitative evaluation of the retinal microvascular network, allowing researchers to visually track vascular degeneration and hemodynamic changes during disease progression *in vivo* (38, 39). This capability provides valuable intermediate biomarkers for use in clinical trials. Retinal organoid technology, on the other hand, generates three-dimensional and multicellular retina-like tissues from induced pluripotent stem cells. This approach largely overcomes the limitations of traditional animal models related to species differences and genetic background variability (40, 41). As a result, retinal organoids have become a practical platform for personalized drug screening and studies of disease mechanisms. The growing adoption of these two tools suggests that RP research is entering a new stage characterized by high-resolution dynamic observation and human-relevant precision modeling.

In addition to OCTA and retinal organoids, several other emerging technologies are also beginning to shape RP research. Artificial intelligence shows strong potential in improving RP diagnosis (42). By training artificial intelligence models on large collections of clinical images and patient data, researchers can achieve automated identification and grading of RP phenotypes, which improves both accuracy and efficiency. Artificial intelligence can also support predictions of disease progression and treatment response, laying the groundwork for more personalized therapeutic planning. Nanomedicine represents another important development. Nanoparticles can act as drug carriers that deliver therapeutic agents to the retina with improved precision and efficiency, increasing bioavailability while reducing unwanted side effects (43). At the same time, nanosensor technology is being actively developed for the detection of ocular and systemic biomarkers. For instance, it has already been employed to visualize metabolic dynamics in retinal cells (44). This capability demonstrates the potential for adapting similar approaches to target RP-specific biomarkers in the future.

### 4.3 Translational bottlenecks and a synergistic framework

Despite strong research activity, RP studies still face several interconnected translational challenges. These include marked genetic and phenotypic heterogeneity, insufficient systems for monitoring disease progression and treatment efficacy, narrow therapeutic time windows, and concerns related to the efficiency and safety of delivery systems (45–49). Importantly, the bibliometric results of this study provide an integrated perspective that may help address these complex issues. Our analysis shows that “gene therapy” and “oxidative stress” are not only frequently occurring keywords but are also closely linked in both the timeline and density analyses. This pattern points to a synergistic therapeutic framework that goes beyond single-modality approaches. Genetic interventions aimed at correcting underlying defects can be viewed as hardware repair, while antioxidant or anti-inflammatory strategies help remodel the pathological microenvironment and act as software optimization. From a biological standpoint, even when a genetic defect is corrected, the retinal microenvironment often remains under chronic oxidative stress and inflammation, which can still impair photoreceptor survival and function (50, 51). Taken together, a framework that integrates genetic correction with microenvironment stabilization holds promise for improving therapeutic outcomes in RP.

Further research into the mechanisms linking gene therapy and cellular stress modulation is essential for developing more effective treatments. It is particularly important to clarify how oxidative stress influences the success of gene therapy and to determine the optimal choice, timing, and dosage of antioxidant interventions to achieve the best combined effect. Understanding these synergistic interactions offers a practical way to address several major translational challenges. First, oxidative stress represents a shared downstream pathway across many genetic subtypes of RP. Targeting this pathway with adjunctive therapies such as antioxidants or anti-inflammatory agents may help overcome genotype-specific limitations and provide broad protective benefits for patients with different mutations. Second, for patients in late stages of disease who have already experienced substantial photoreceptor loss, genetic correction alone often has a limited impact. Combining it with neuroprotective or microenvironment-modulating approaches may help preserve remaining cells and slow disease progression, thereby extending therapeutic benefit to a wider patient population. Third, adding antioxidant strategies may reduce local inflammatory responses triggered by gene therapy vectors and improve the cellular environment. This could enhance the survival and functional stability of treated cells and improve overall safety. This synergistic framework represents not only a novel treatment concept but also a structured way to manage the complexity of RP. Future studies should focus on defining the molecular basis of this synergy and identifying optimal combination strategies, including drug selection, timing, and dosage balance. Well-designed clinical trials will be needed to confirm whether this approach can overcome the limits of current single treatments and support a shift from isolated advances toward broader and more sustained benefits in RP care.

## 5 Research limitations and prospects

Although bibliometric analysis has successfully identified research hotspots and trends in RP, several challenges and limitations remain. These include data inconsistencies across various databases, variations in research methodologies employed by different studies, and difficulties in comparing findings from studies with disparate focus areas, all of which can hinder the accuracy and generalizability of the analysis. Furthermore, the analysis is constrained by data coverage and timeliness. Since the analysis primarily relies on peer-reviewed literature, preprints and ongoing trials are often not represented, potentially obscuring emerging trends. Additionally, due to the rapid pace of the field, a significant time lag exists between scientific advances and their incorporation into datasets, meaning the analysis may not capture the very latest shifts in research direction.

Looking forward, future bibliometric analyses in this field should aim to reduce these limitations by drawing on a wider range of data sources, including preprint servers, conference abstracts, patents, and clinical trial registries. Bringing together these near real-time data streams can help reduce publication delays and better capture recent advances, such as newly launched gene therapy trials, before they appear in traditional journals. Furthermore, adopting standardized data extraction protocols and more advanced natural language processing methods will be important for addressing data inconsistencies and improving the reliability of comparisons across databases. By moving toward a more flexible and inclusive analytical framework, future studies can offer researchers and clinicians timely and detailed insights into the continuously evolving landscape of RP research.

## 6 Conclusion and implications

In summary, this study uses a bibliometric perspective to outline the dynamic development of RP research. The field is rapidly shifting from a traditional focus on genetic discovery to a more forward-looking direction driven by technological advances, strong multidisciplinary integration, and an emphasis on systemic intervention strategies. Beyond confirming the key role of gene therapy, the analysis highlights the growing importance of a synergistic approach that combines cellular stress modulation, such as oxidative stress control, with genetic repair. This emerging paradigm represents a new research frontier and a possible turning point for future breakthroughs. These findings have practical implications for funding agencies when defining priority areas, for research teams when choosing cross-disciplinary collaborations, and for clinicians when designing next-generation combination therapies. RP research is likely to continue progressing under the combined principles of precision correction and systemic support, moving steadily toward a more effective and personalized era of treatment.

## Data Availability

The original contributions presented in this study are included in this article/supplementary material, further inquiries can be directed to the corresponding author.

## References

[1] Liu W Liu S Li P Yao K. Retinitis pigmentosa: progress in molecular pathology and biotherapeutical strategies. *Int J Mol Sci.* (2022) 23:4883. 10.3390/ijms23094883 35563274 PMC9101511

[2] Chen Y Lin M Zhuang D. Wastewater treatment and emerging contaminants: bibliometric analysis. *Chemosphere.* (2022) 297:133932. 10.1016/j.chemosphere.2022.133932 35149018

[3] Huang Y Gong D Dang K Zhu L Guo J Yang W The applications of anterior segment optical coherence tomography in glaucoma: a 20-year bibliometric analysis. *PeerJ.* (2024) 12:e18611. 10.7717/peerj.18611 39619196 PMC11608565

[4] Vought V Vought R Herzog A Mothy D Shukla J Crane AB Evaluating research activity and NIH-Funding among academic ophthalmologists using relative citation ratio. *Semin Ophthalmol.* (2025) 40:39–43. 10.1080/08820538.2024.2391838 39149972

[5] Zhu J Liang Q He S Wang C Lin X Wu D Research trends and hotspots of neurodegenerative diseases employing network pharmacology: a bibliometric analysis. *Front Pharmacol.* (2023) 13:1109400. 10.3389/fphar.2022.1109400 36712694 PMC9878685

[6] Mao Y Zhao K Chen N Fu Q Zhou Y Kong C A 2-decade bibliometric analysis of epigenetics of cardiovascular disease: from past to present. *Clin Epigenet.* (2023) 15:184. 10.1186/s13148-023-01603-9 38007493 PMC10676610

[7] Ioannidis JPA Baas J Klavans R Boyack KW. A standardized citation metrics author database annotated for scientific field. *PLoS Biol.* (2019) 17:e3000384. 10.1371/journal.pbio.3000384 31404057 PMC6699798

[8] Rodrigues M Antunes JA Miguéis V. Aligning priorities: A Comparative analysis of scientific and policy perspectives on municipal solid waste management. *Waste Manag.* (2024) 193:70–83. 10.1016/j.wasman.2024.11.031 39642404

[9] van Eyk HJ Hooiveld MH Van Leeuwen TN Van der Wurff BL De Craen AJ Dekker FW Scientific output of Dutch medical students. *With Teach.* (2010) 32:231–5. 10.3109/01421591003596592 20218838

[10] Silva SC Ferreira ICFR Dias MM Barreiro MF. Microalgae-derived pigments: a 10-year bibliometric review and industry and market trend analysis. *Molecules.* (2020) 25:3406. 10.3390/molecules25153406 32731380 PMC7435790

[11] Chen C. CiteSpace II: detecting and visualizing emerging trends and transient patterns in scientifc literature. *J Am Soc Inform Sci Technol.* (2006) 57:359–77. 10.1002/asi.20317

[12] van Eck NJ Waltman L. Software survey: VOSviewer, a computer program for bibliometric mapping. *Scientometrics.* (2010) 84:523–38. 10.1007/s11192-009-0146-3 20585380 PMC2883932

[13] Ellegaard O Wallin JA. The bibliometric analysis of scholarly production: how great is the impact? *Scientometrics.* (2015) 105:1809–31. 10.1007/s11192-015-1645-z 26594073 PMC4643120

[14] Yu L Xu C Zhang M Zhou Y Hu Z Li L Top 100 cited research on COVID-19 vaccines: a bibliometric analysis and evidence mapping. *Hum Vaccin Immunother.* (2024) 20:2370605. 10.1080/21645515.2024.2370605 38977415 PMC11232646

[15] Gu SM Thompson DA Srikumari CR Lorenz B Finckh U Nicoletti A Mutations in RPE65 cause autosomal recessive childhood-onset severe retinal dystrophy. *Nat Genet.* (1997) 17:194–7. 10.1038/ng1097-194 9326941

[16] Santos A Humayun MS de Juan E Jr. Greenburg RJ Marsh MJ Klock IB Preservation of the inner retina in retinitis pigmentosa. a morphometric analysis. *Arch Ophthalmol.* (1997) 115:511–5. 10.1001/archopht.1997.01100150513011 9109761

[17] Milam AH Li ZY Fariss RN. Histopathology of the human retina in retinitis pigmentosa. *Prog Retin Eye Res.* (1998) 17:175–205. 10.1016/s1350-9462(97)00012-8 9695792

[18] Weng J Mata NL Azarian SM Tzekov RT Birch DG Travis GH. Insights into the function of Rim protein in photoreceptors and etiology of Stargardt’s disease from the phenotype in abcr knockout mice. *Cell.* (1999) 98:13–23. 10.1016/S0092-8674(00)80602-9 10412977

[19] Gal A Li Y Thompson DA Weir J Orth U Jacobson SG Mutations in MERTK, the human orthologue of the RCS rat retinal dystrophy gene, cause retinitis pigmentosa. *Nat Genet.* (2000) 26:270–1. 10.1038/81555 11062461

[20] Mears AJ Kondo M Swain PK Takada Y Bush RA Saunders TL Nrl is required for rod photoreceptor development. *Nat Genet.* (2001) 29:447–52. 10.1038/ng774 11694879

[21] Chang B Hawes NL Hurd RE Davisson MT Nusinowitz S Heckenlively JR. Retinal degeneration mutants in the mouse. *Vision Res.* (2002) 42:517–25. 10.1016/s0042-6989(01)00146-8 11853768

[22] Marc RE Jones BW Watt CB Strettoi E. Neural remodeling in retinal degeneration. *Prog Retin Eye Res.* (2003) 22:607–55. 10.1016/s1350-9462(03)00039-9 12892644

[23] Humayun MS Weiland JD Fujii GY Greenberg R Williamson R Little J Visual perception in a blind subject with a chronic microelectronic retinal prosthesis. *Vision Res.* (2003) 43:2573–81. 10.1016/s0042-6989(03)00457-7 13129543

[24] MacLaren RE Pearson RA MacNeil A Douglas RH Salt TE Akimoto M Retinal repair by transplantation of photoreceptor precursors. *Nature.* (2006) 444:203–7. 10.1038/nature05161 17093405

[25] Chen J Patil S Seal S McGinnis JF. Rare earth nanoparticles prevent retinal degeneration induced by intracellular peroxides. *Nat Nanotechnol.* (2006) 1:142–50. 10.1038/nnano.2006.91 18654167

[26] Bi A Cui J Ma YP Olshevskaya E Pu M Dizhoor AM Ectopic expression of a microbial-type rhodopsin restores visual responses in mice with photoreceptor degeneration. *Neuron.* (2006) 50:23–33. 10.1016/j.neuron.2006.02.026 16600853 PMC1459045

[27] Lin JH Li H Yasumura D Cohen HR Zhang C Panning B IRE1 signaling affects cell fate during the unfolded protein response. *Science.* (2007) 318:944–9. 10.1126/science.1146361 17991856 PMC3670588

[28] Zrenner E Bartz-Schmidt KU Benav H Besch D Bruckmann A Gabel VP Subretinal electronic chips allow blind patients to read letters and combine them to words. *Proc Biol Sci.* (2011) 278:1489–97. 10.1098/rspb.2010.1747 21047851 PMC3081743

[29] Russell S Bennett J Wellman JA Chung DC Yu ZF Tillman A Efficacy and safety of voretigene neparvovec (AAV2-hRPE65v2) in patients with RPE65-mediated inherited retinal dystrophy: a randomised, controlled, open-label, phase 3 trial. *Lancet.* (2017) 390:849–60. 10.1016/S0140-6736(17)31868-8 28712537 PMC5726391

[30] Xiao L Yang YJ Liu Q Peng J Yan JF Peng QH. Visualizing the intellectual structure and recent research trends of diabetic retinopathy. *Int J Ophthalmol.* (2021) 14:1248–59. 10.18240/ijo.2021.08.18 34414092 PMC8342278

[31] Dayma K Rajanala K Upadhyay A. Stargardt’s disease: molecular pathogenesis and current therapeutic landscape. *Int J Mol Sci.* (2025) 26:7006. 10.3390/ijms26147006 40725253 PMC12295471

[32] Gumerson JD Alsufyani A Yu W Lei J Sun X Dong L Restoration of RPGR expression in vivo using CRISPR/Cas9 gene editing. *Gene Ther.* (2022) 29:81–93. 10.1038/s41434-021-00258-6 34257417 PMC8856954

[33] Bakondi B Lv W Lu B Jones MK Tsai Y Kim KJ In vivo CRISPR/Cas9 gene editing corrects retinal dystrophy in the S334ter-3 rat model of autosomal dominant retinitis pigmentosa. *Mol Ther.* (2016) 24:556–63. 10.1038/mt.2015.220 26666451 PMC4786918

[34] Jang A Petrova B Cheong TC Zawadzki ME Jones JK Culhane AJ Choroid plexus-CSF-targeted antioxidant therapy protects the brain from toxicity of cancer chemotherapy. *Neuron* (2022) 110:3288–301.e8. 10.1016/j.neuron.2022.08.009. 36070751 PMC9588748

[35] Miraldi Utz V Coussa RG Antaki F Traboulsi EI. Gene therapy for RPE65-related retinal disease. *Ophthalmic Genet.* (2018) 39:671–7. 10.1080/13816810.2018.1533027 30335549

[36] Yang Y Han X Shen J Zhu Z Lu P. Targeted metabolomics reveals dysregulated tryptophan metabolism in retinitis pigmentosa. *Exp Eye Res.* (2025) 260:110624. 10.1016/j.exer.2025.110624 40907860

[37] Yang M Qiu R Jin X Yao S Wang W Liu J Proteomics identifies multiple retinitis pigmentosa associated proteins involved in retinal degeneration in a mouse model bearing a Pde6b mutation. *Sci Rep.* (2024) 14:22090. 10.1038/s41598-024-72821-1 39333705 PMC11437026

[38] Koyanagi Y Murakami Y Funatsu J Akiyama M Nakatake S Fujiwara K Optical coherence tomography angiography of the macular microvasculature changes in retinitis pigmentosa. *Acta Ophthalmol.* (2018) 96:e59–67. 10.1111/aos.13475 28561452

[39] Takagi S Hirami Y Takahashi M Fujihara M Mandai M Miyakoshi C Optical coherence tomography angiography in patients with retinitis pigmentosa who have normal visual acuity. *Acta Ophthalmol.* (2018) 96:e636–42. 10.1111/aos.13680 29498230 PMC6175316

[40] Seah I Goh D Banerjee A Su X. Modeling inherited retinal diseases using human induced pluripotent stem cell derived photoreceptor cells and retinal pigment epithelial cells. *Front Med.* (2024) 11:1328474. 10.3389/fmed.2024.1328474 39011458 PMC11246861

[41] Ma C Jin K Jin ZB. Generation of human patient iPSC-derived retinal organoids to model retinitis pigmentosa. *J Vis Exp.* (2022) 184:e64045. 10.3791/64045 35786611

[42] Parmar UPS Surico PL Singh RB Romano F Salati C Spadea L Artificial Intelligence (AI) for early diagnosis of retinal diseases. *Medicina.* (2024) 60:527. 10.3390/medicina.60040527 38674173 PMC11052176

[43] Tawfik M Chen F Goldberg JL Sabel BA. Nanomedicine and drug delivery to the retina: current status and implications for gene therapy. *Naunyn Schmiedebergs Arch Pharmacol.* (2022) 395:1477–507. 10.1007/s00210-022-02287-3 36107200 PMC9630211

[44] Calbiague García V Chen Y Cádiz B Wang L Paquet-Durand F Schmachtenberg O. Imaging of lactate metabolism in retinal Müller cells with a FRET nanosensor. *Exp Eye Res.* (2023) 226:109352. 10.1016/j.exer.2022.109352 36528083

[45] Ferrari S Di Iorio E Barbaro V Ponzin D Sorrentino FS Parmeggiani F. Retinitis pigmentosa: genes and disease mechanisms. *Curr Genom.* (2011) 12:238–49. 10.2174/138920211795860107 22131869 PMC3131731

[46] Pierce EA Bennett J. The status of RPE65 gene therapy trials: safety and efficacy. *Cold Spring Harb Perspect Med.* (2015) 5:a017285. 10.1101/cshperspect.a017285 25635059 PMC4561397

[47] Garafalo AV Cideciyan AV Héon E Sheplock R Pearson A WeiYang Yu C Progress in treating inherited retinal diseases: early subretinal gene therapy clinical trials and candidates for future initiatives. *Prog Retin Eye Res.* (2020):100827. 10.1016/j.preteyeres.2019.100827 31899291 PMC8714059

[48] Kotterman MA Schaffer DV. Engineering adeno-associated viruses for clinical gene therapy. *Nat Rev Genet.* (2014) 15:445–51. 10.1038/nrg3742 24840552 PMC4393649

[49] Mingozzi F High KA. Immune responses to AAV in clinical trials. *Curr Gene Ther.* (2011) 11:321–30. 10.2174/156652311796150354 21557723

[50] Lu Z Liu S Morales MG Whitlock A Ramkumar R Ramkumar HL. Retinal BMI1 expression preserves photoreceptors in sodium-iodate-induced oxidative stress models. *Int J Mol Sci.* (2025) 26:5907. 10.3390/ijms26125907 40565365 PMC12193584

[51] Bighinati A Adani E Stanzani A D’Alessandro S Marigo V. Molecular mechanisms underlying inherited photoreceptor degeneration as targets for therapeutic intervention. *Front Cell Neurosci.* (2024) 18:1343544. 10.3389/fncel.2024.1343544 38370034 PMC10869517

